# Quality of life and mental health of children with rare congenital surgical diseases and their parents during the COVID-19 pandemic

**DOI:** 10.1186/s13023-021-02129-0

**Published:** 2021-11-27

**Authors:** Mareike Fuerboeter, Johannes Boettcher, Claus Barkmann, Holger Zapf, Rojin Nazarian, Silke Wiegand-Grefe, Konrad Reinshagen, Michael Boettcher

**Affiliations:** 1 Department of Pediatric Surgery, University Medical Center Hamburg-Eppendorf, Martinistrasse 52, 20246 Hamburg, Germany; 2 Department of Child and Adolescent Psychiatry, Psychosomatics and Psychotherapy, University Medical Center Hamburg-Eppendorf, Martinistrasse 52, 20246 Hamburg, Germany; 3 Department of Pediatric Surgery, University Medical Center Mannheim, Theodor-Kutzer-Ufer 1-3, 68167 Mannheim, Germany

**Keywords:** Quality of life, Health-related quality of life, Mental health, Rare diseases, Parents, Pediatric surgery

## Abstract

**Background:**

COVID-19 has affected our society at large, particularly vulnerable groups, such as children suffering from rare diseases and their parents. However, the psychosocial influences of COVID-19 on these have yet to be investigated. As such, the study’s goal was to evaluate the health-related quality of life (HRQoL), quality of life (QoL), and mental health of children with rare congenital surgical diseases and their parents during the COVID-19 pandemic and lockdown measures.

**Methods:**

A survey of *n* = 210 parents of children with rare congenital surgical diseases and a control group of *n* = 88 parents of children without rare diseases was conducted cross-sectionally between April 2020 to April 2021. Data on HRQoL, QoL, and mental health was collected using standardized psychometric questionnaires for children and parents presenting to the pediatric surgery department at a university hospital.

**Results:**

Mothers of children with rare pediatric surgical diseases showed significantly lower QoL and significantly higher impairment in mental health than a control group and norm data. For fathers, this was solely the case for their QoL. Children’s parent-reported HRQoL and mental health were partially impaired. Social and disease-specific risk factors of the respective outcomes in affected families were identified through regression analysis models.

**Conclusion:**

Parents of children with rare diseases report severe psychosocial impairment regarding themselves and their children during the COVID-19 pandemic. Therefore, affected families should receive attention and supportive care in the form of a family-center approach to alleviate the additional burden of the COVID-19 pandemic.

**Supplementary Information:**

The online version contains supplementary material available at 10.1186/s13023-021-02129-0.

## Background

Children with rare diseases and their parents have often been an overlooked population within the healthcare system and healthcare research [1]. Even though the diagnoses of rare diseases are very heterogeneous, the burden on the affected patients and their families appears very similar, as the majority of rare diseases are most severe, chronic, progressive, arise from genetic causes, and associated with a shortened life expectancy [2, 3]. However, even in the best of times, children with rare diseases and their parents report that they face substantial care deficiencies and unmet clinical needs because of their disease [4]. Despite significant progress in meeting these unmet needs for affected patients and their parents, the COVID-19 pandemic has dismantled this progress [5]. Especially children with rare diseases and their parents potentially face severe health threats during the COVID-19 pandemic, in addition to the inadequate care and support they already met before [6].

Rare diseases are defined by a prevalence of less than 1:2000 [7]. According to estimates, there are more than 7000 different rare diseases [8]. The proportion of people diagnosed with a rare disease is estimated to be around 30 million people in Europe and 25 million in the US [2]. One population struggling with the burden of a rare disease are children and adolescents with pediatric conditions requiring surgical treatment, namely anorectal malformations, biliary atresia, congenital diaphragmatic hernia, esophageal atresia, or Hirschsprung’s disease. These conditions are either (1) congenital structural anomalies that are present at birth or (2) arise in early childhood and require surgical treatment within the first days or months of life [9]. Fortunately, many conditions can be treated with a single surgery, but others might require multiple surgeries and result in long-term illness and life-long afflictions as well as chronic disability [9–11].

Challenges faced by patients with rare diseases and their parents are manifold and include cognitive, emotional, and physical impairments [12], leading to a decreased quality of life (QoL) and mental health [13, 14]. The concept of QoL can be described as “the individuals’ perception of their position in life in the context of the culture and value systems in which they live, in relation to their goals, expectations, standards and concerns” [15]. Mental health, in contrast, can be defined as the “flexibility and ability to cope with adverse life events and function in social roles” [16]. Considering rare pediatric diseases in the diathesis-stress model [17], the interaction of individual vulnerabilities characteristics of the rare disease and its consequences can explain the lower QoL and mental health in both the affected children and their parents.

Correspondingly, previous studies suggest that parents of children with rare congenital surgical diseases score significantly lower on QoL [18–21] and mental health assessments [22, 23]. Moreover, studies involving children with rare congenital surgical diseases have shown a significant reduction in parent-reported health-related quality of life (HRQoL) compared to healthy controls [24–28]. In addition to these findings, research has also shown that children with such rare conditions are at risk for developing emotional and behavioral problems [29–32].

The COVID-19 pandemic and the resulting lockdown measures have been suggested to affect 1.6 billion children, and a recent study found that children, in particular, are affected by the COVID-19 pandemic [33]. Although COVID-19 is unlikely to cause severe disease in children, up to 10% of children might show symptoms for weeks [34]. More important, children and adolescents face massive restrictions in their daily lives, including school closures, home confinement, and social distancing rules. As psychosocial parameters of children with rare congenital surgical diseases and their parents during the COVID-19 pandemic and lockdown measures have not been evaluated, the current study aimed to assess the HRQoL and the mental health of children with rare congenital surgical diseases and their parents during the COVID-19 pandemic and lockdown measures. Therefore, the following research questions were addressed. (1) Are there differences in the distribution of HRQoL, QoL, and mental health between affected and unaffected patients and their parents during the COVID-19 pandemic? (2) Are there differences in the distribution of HRQoL, QoL, and mental health between affected patients and their parents during the COVID-19 pandemic and norm values? (3) To what extent are the psychosocial outcomes of HRQoL, QoL, and mental health of affected children, their mothers, and fathers associated? (4) What factors can explain the variances in the respective outcome values in the index group?

We expect the overall QoL to be lower and the overall psychological distress of affected parents to be higher compared to the control group and norm values. Moreover, we expect lower overall parent-reported HRQoL and higher parent-reported overall psychological distress in children with rare congenital surgical diseases than the control group and norm values using mental health metrics. We expect significant associations between psychosocial outcomes of affected children, their mothers, and fathers. Moreover, we expect that affected children and their parents with low education, high level of care, and a shorter time interval from the last operation to be impacted significantly more. This study provides information on specific problem areas of the relevant constructs and identifies whether children and their parents are at risk for impaired QoL and mental health.

## Methods

### Study design

In this cross-sectional observational study with two groups, the index group was defined as families of children with a rare congenital surgical disease. At the same time, the control group comprised families of children undergoing routine surgical procedures without a prior diagnosed rare disease. The index and control group were recruited during the COVID-19 pandemic between April 2020 and April 2021 and were required to answer a set of standardized psychometric questionnaires. In addition, to compare the index group data with data collected before the COVID-19 pandemic, appropriate population-based normative data were used. To fully implement a “COVID-19 Yes–No” intervention variable, the same data would have to have been collected for both groups before the pandemic, which was not possible. Therefore, norm data of the outcome questionnaires were used here, or, alternatively, a narrative comparison to existing studies was chosen. Unlike the primary data of the two groups under pandemic conditions, the norm data are secondary data based on different samples and time points. Nevertheless, these norm values provide a rough approximation of the distribution of the outcomes under “non-pandemic conditions.” The study received ethical approval from the Medical Chamber Hamburg (PV7161) and was preregistered at ClinicalTrials.gov (NCT04382820).

### Variables and instruments

#### Parental quality of life

The Ulm Quality of Life Inventory for Parents (ULQIE) was designed for parents of chronically ill children and consists of 29 items, which are answered on a five-point rating scale [35]. Five respective subscales measure (1) physical and daily functioning (seven items), (2) satisfaction with the family (six items), (3) emotional distress (four items), (4) self-development (four items), and (5) well-being (four items). Four other items have no scale assignment. Negative items were reversed-scored so that better QoL complies with higher scores. The ULQIE has been shown to provide reliable psychometric properties and norm data for parents of chronically ill children suffering from various diseases [35].

#### Parental mental health

The Brief Symptom Inventory (BSI) was used to evaluate the parental mental health status [36]. The BSI includes 53 items covering (1) somatization (seven items), (2) compulsivity (six items), (3) interpersonal sensitivity (four items), (4) depression (six items), (5) anxiety (six items), (6) hostility (five items), (7) phobic fear (five items), (8) paranoid thinking (five items), and (9) psychoticism (5 items). There are five more items that are not assigned to any scale. Higher BSI scores indicate higher psychological distress. Furthermore, the General Symptom Index (GSI) was calculated as a global index of psychological distress, ranging from 0 to 36. Additionally, sum scores were converted into T-scores according to the normative population of the test manual. GSI T-scores greater or equal to 63 or two or more subscales are defined as clinically significant [36]. The German version of the BSI has been found to assess psychometric properties of individuals in a reliable and valid fashion, and thus it has been able to provide normative data for the German general population separated by gender [37].

#### Children’s health related quality of life

The Pediatric Quality of Life Inventory™ Short Form 15 (PedsQL™ 4.0 SF15) was used to assess HRQoL in children and adolescents aged 2 to 18 years [38]. The parent-report measure includes four subscales encompassing (1) physical functioning (five items), (2) emotional functioning (four items), (3) social functioning (three items), and (4) school functioning (three items). Raw scores are then converted into a standardized 0 to 100 scale according to the manual, with higher scores representing greater HRQoL. A psychosocial and physical score as well as a total score was calculated to illustrate overall HRQoL. The German version of the PedsQL 4.0 SF-15 has shown adequate psychometric properties [39]. Normative values of the PedsQL 4.0 SF-15 were obtained from previously conducted validation studies [38]. To differentiate good HRQoL from notable impairment in HRQoL, we used a PedsQL score of $\le \hspace{0.17em}$65 as a cut-off score [40, 41].

#### Children’s mental health

The Strengths and Difficulties Questionnaire (SDQ) assesses emotional and behavioral status, as well as prosocial behavior [42]. The instrument comprises five subscales with five items each, including (1) emotional symptoms, (2) conduct problems, (3) hyperactivity, (4) peer problems, and (5) prosocial behavior. An additional total score can be calculated. In the present study, the German version of the parent-reported SDQ for children and adolescents aged 3–16 years was used [42]. Higher scores represent greater problems on all subscales, except for the subscale prosocial behavior, where lower scores correspond to more difficulties in prosocial behavior. This version of the SDQ has shown acceptable internal consistencies and provides population-based norm data [43, 44]. Additionally, cut-off values were used to differentiate between normal and clinical relevant range (borderline and abnormal) of scores [45].

#### Socio-demographic and clinical variables

Participants completed a study-specific questionnaire including sex, age, number of siblings, marital status, education, and employment status of the parents. Clinical variables included time since the initial diagnosis and last surgery, number of surgeries, patient-level of care, type of rare condition, and comorbid VACTERL-association.

### Sample

#### Index group

Inclusion criteria for the index group were as follows: (1) age < 21 years, (2) previous diagnosis of a rare congenital surgical disease, including anorectal malformations, biliary atresia, congenital diaphragmatic hernia, esophageal atresia, or Hirschsprung’s disease. Severe physical, mental or cognitive impairments were set as exclusion criteria, as patient participation would have been impossible or unreasonable. Signed informed consent was given by the patients’ parents. Participants were allowed to withdraw from the study at any given time. All participating children met the European Commission definition for a rare disease [7], and all diagnoses were verified by medical personnel before enrollment in the study.

In total, 342 families with children with rare congenital surgical diseases were identified between 2012 and 2020 in the operative registry of the Clinic of Pediatric Surgery of the University Medical Center Hamburg Eppendorf, Hamburg, Germany (Universitätsklinikum Hamburg-Eppendorf – UKE).

Out of the 342 families, 13 were excluded due to patient’s death, and 37 were excluded due to our inability to contact the families. As such, 292 families were asked to participate in this study, with 84 families denying participation straight away, resulting in handing out the questionnaire to 208 families. Out of the 208 families, the response rate was 54.8% for families of rare diseased children. A total of 210 parents completed the parental QoL and mental health assessment, with an almost equal distribution of gender (109 mothers and 101 fathers). Parent-ratings regarding the HRQoL and mental health measures were provided for 114 children: 96 (84.2%) of all parent-ratings were answered by both parents, while 13 (11.4%) were answered by the mother alone and five (4.4%) by the father.

#### Control group

**Fig. 1:**
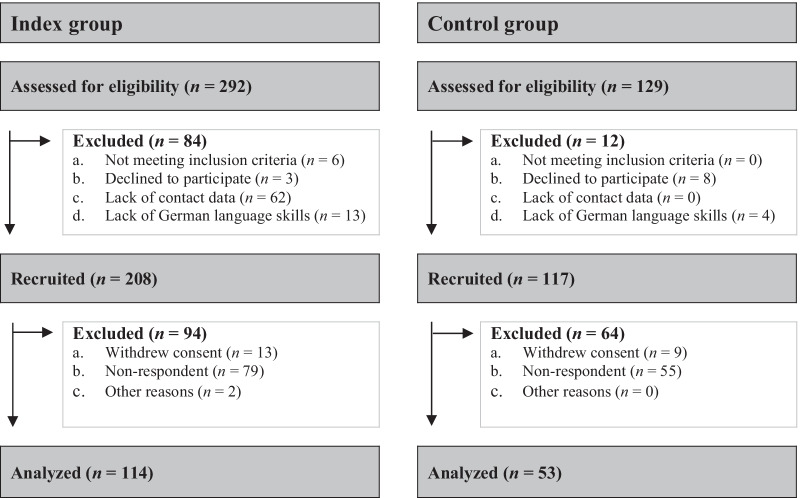
CONSORT flow diagram

Inclusion criteria for the control group were families of children without prior diagnosis or signs of rare chronic symptoms or conditions. In total, 129 families were assessed for eligibility. Out of these, eight families declined to participate (*n* = 8), and four families were excluded due to a lack of German language skills (*n* = 4), so 117 families received the questionnaire. Overall, 53 families (45.3% response rate) answered the questionnaires before obtaining written consent. In the control group, 88 parents completed the questionnaires about parental QoL and mental health (52 mothers and 36 fathers). Parent ratings regarding the HRQoL and mental health measures were provided for 53 children. 35 (66.0%) of all parent ratings were answered by both parents, while 17 (32.1%) were answered solely by the mother and one (1.9%) by a father. Figure 1 shows the CONSORT flow diagram.

### Statistics

For descriptive issues, frequencies, means, standard deviations, and bivariate tests (chi-square tests) were used. Differences between the index group and normative reference scores were investigated using one-sample *t* tests. Differences between the index and control group were analyzed using Welch’s *t* test. Differences between mothers and fathers were studied using the *t* test for dependent samples, while an Intraclass correlation (ICC) was conducted between mothers and fathers’ parent reports. Pearson correlations were used to investigate the bivariate associations between psychosocial outcomes. In order to define predictors of psychosocial outcomes, multiple linear regression models were conducted. To indicate the size of the effect, Cohen’s d and Cramer’s V were calculated. Statistical significance was set at *p *$\le \hspace{0.17em}$0.05 (two-tailed). To address a possible bias due to missing data, multiple imputation using the Markov Chain Monte Carlo (MCMC) approach was used. Statistical analyses were conducted using SPSS Statistics 26 and Graphpad Prism 9.

## Results

### Characteristics of the study populations

Table 1 shows the sociodemographic and disease characteristics of the participating families in the index and control group. Regarding the child’s age, there was a medium sized difference between participants (*M* = 4.2, *SD* = 3.33) and non-participants (*M* = 6.5, *SD* = 3.96) in the index group (*d* = 0.61, *p* < 0.001). However, the child’s gender between participants (female = 44, male = 70) and non-participants (female = 123, male = 107) did not differ (*Cramer’s V* = 0.14, *p* = 0.137,). Finally, regarding the disease-groups, no difference between participants (anorectal malformation = 30, biliary atresia = 14, congenital diaphragmatic hernia = 14, esophageal atresia = 27, Hirschsprung’s disease = 29) and non-participants (anorectal malformation = 65, biliary atresia = 20, congenital diaphragmatic hernia = 27, esophageal atresia = 40, Hirschsprung’s disease = 76) could be found (*Cramer’s V* = 0.11, *p* = 0.394). For the comparison of the index and the control group, the groups had overall similar demographics. No relevant difference was found between the parents of the index and the control group for the age of the affected child (*d* = 0.06, *p* = 0.806,), mothers (*d* = − 0.07, *p* = 0.694,), and fathers (*d* = − 0.07, *p* = 0.548). Gender distribution of the children did not differ between the index and control group (*Cramer’s V* = 0.05, *p* = 0.604,). Moreover, there were no differences in terms of the gender of the parents, marital status, education level, employment, and social support of the families. Subgroup analyses revealed that no significant difference occurred in any HRQoL, QoL, and mental health subscale between the different disease groups and whether there was a partial lockdown taking place.

**Table 1:** Sociodemographic and disease characteristics of the index and control group

Characteristics	Index group (*n* = 114 families)		Control group (*n* = 53 families)	
	*M*	*SD*	*M*	*SD*
Patient’s age (years)	4.2	3.33	4.3	3.25
Mother’s age (years)	37.4	5.98	37.1	5.50
Father’s age (years)	40.3	6.15	39.7	6.49
Number of children in family	2.0	0.78	1.6	0.69
Number of surgeries due to disease	4.4	4.21	–	–
Time since last surgery (years)	2.6	2.51	*–*	–
Time since first surgery (years)	3.8	3.04	*–*	–

Parents	*n*	%	*n*	%
Parent’s gender (mothers/fathers)	109/101	95.6/88.6	52/36	96.0/64.0
Marital status (mothers/fathers)				
Married/living together	98/92	89.9/91.1	43/32	82.7/88.9
Single	9/7	8.3/6.9	6/2	11.5/5.6
Divorced	2/2	1.8/2.0	3/1	5.8/2.8
Not stated	0/0	0.0/0.0	0/1	0.0/2.8
Education (mothers/fathers)				
Lower-middle education	32/42	29.4/41.6	9/6	17.3/16.7
Higher education	69/54	63.3/53.5	41/29	78.8/80.6
Not stated	8/5	7.3/5.0	2/1	3.8/2.8
Employment^a^ (mothers/fathers)				
Fully employed	11/94	10.1/93.1	8/34	15.4/94.4
Partly employed	54/4	49.5/4.0	24/1	46.2.2/2.8
No employment	42/3	38.5/3.0	20/1	38.5/2.8
Not stated	2/0	1.8/0.0	0/0	0.0/0.0

Patients	*n*	%	*n*	%
Patient’s gender				
Female	44	38.6	24	45.3
Male	70	61.4	29	54.7
Patient receives level of care^b^				
Yes	43	37.7	–	–
No	71	62.3	–	–
Patient rare disease				
Anorectal malformation	30	26.3	–	–
Biliary atresia	14	12.3	–	–
Congenital diaphragmatic hernia	14	12.3	–	–
Esophageal atresia	27	23.7	-	-
Hirschsprung’s disease	29	25.4	–	–
VACTERL association				
Yes	16	14.0	–	–
No	98	86.0	–	–

^a^Refers to the last 12 months

^b^Refers to the decision for the classification in the care insurance according to the German long-term care insurance

### Differences in psychosocial variables between families of children with rare diseases, families without rare diseases, and norm values

Table 2 shows the distribution of overall parental QoL and mental health, as well as overall parent-reported HRQoL and mental health of the child from the perspective of both mothers and fathers in the index and control group. In addition, a comparison to norm data is given. The mothers and fathers in the index group had significantly lower scores on the overall QoL than mothers and the fathers in the control group. Regarding mental health, mothers but not fathers showed significantly higher scores than mothers and fathers in the control group. Parent-reported HRQoL was significantly lower in both mothers and fathers of the index group compared to the control group, whereas only a significant difference was found for parent-reported mental health in mothers, but not fathers between both groups. Effect sizes ranged from trivial to large.

**Table 2:** Distribution of parental QoL and mental health, and parent-reported HRQoL and mental health for the index group, the control group, and norm data of the ULQIE, BSI, PedsQL SF15, and SDQ

	Index group (a)		Control group (b)		Norm data (c)		Differences	Effect size a versus b	Effect size a versus c
	*M*	SD	*M*	SD	*M*	SD			
Mothers									
QoL	2.3	0.31	2.6	0.52	2.6	0.53	a < b***; a < c***	− 0.82	− 1.17
Mental health	0.5	0.48	0.3	0.24	0.4	0.23	a > b***; a > c**	0.52	0.27
Children’s HRQoL	81.5	13.79	87.7	9.80	86.1	11.2	a < b***; a < c**	− 0.49	− 0.33
Children’s mental health	11.3	3.65	10.1	2.96	8.5	7.22	a > b*; a > c***	0.33	0.75
Fathers									
QoL	2.4	0.30	2.8	0.60	2.6	0.53	a < b***; a < c***	− 1.02	− 0.96
Mental health	0.3	0.28	0.3	0.37	0.3	0.23	a > b; a < c	0.07	− 0.05
Children’s HRQoL	85.0	11.91	91.2	8.37	86.1	11.2	a < b***; a < c	− 0.56	− 0.09
Children’s mental health	11.1	4.01	10.2	3.53	8.5	7.22	a > b; a > c***	0.24	0.65

Comparison between groups is assessed with Welch *t* test and one-sample *t* test

*QoL* quality of life, *HRQoL* health-related quality of life, *ULQIE* Ulm quality of life scale, *BSI* *GSI* brief symptom inventory—global severity index, *PedsQL SF15* Pediatric Quality of Life Inventory Short Form 15, *SDQ* strength and difficulties questionnaire

*p ≤ .05; **p ≤ .01; ***p ≤ .001, *d* = Cohen’s *d*

Compared to norm data of parents of children with chronic diseases, mothers and fathers of the index group showed significantly lower scores on overall QoL. For mental health, mothers but not fathers of the index group showed significantly higher overall mental health scores than norm data of a healthy population. Parent-reported HRQoL was significantly lower in mothers but not fathers of the index group than norm data of healthy children, albeit significant differences were found for parent-reported mental health between the index group and norm data of German children. Effect sizes again ranged from trivial to large. A more nuanced view of the individual subscales of the corresponding outcome variables is presented in Additional file 1: Table S1–S4.

Post-hoc comparison of gender differences in the index group showed that mothers compared to fathers had significantly lower scores on overall QoL (*d* = − 0.25, *p* = 0.029,) and significantly higher scores on overall mental health (*d* = 0.52, *p* < 0.001). Subsequent analyses indicated that the concordance of parent-report ratings of mothers and fathers overall QoL (ICC = 0.79) and mental health (ICC = 0.65) in the index group showed fair to good reliability.

### Proportion of parents and affected children at risk for psychosocial impairment

**Fig. 2:**
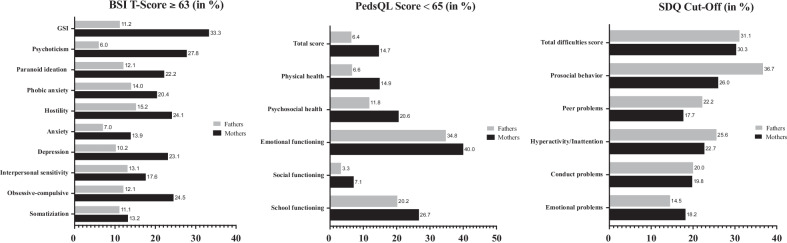
Percentages of parents and children in the index group at risk for psychosocial impairment

Figure 2 shows the percentages of parents and children in the index group at risk for psychosocial impairment. Mothers showed generally higher impairment in all mental health subscales and the GSI compared to fathers. Concerning the HRQOL of the children, it is evident that the area of emotional functioning in both mothers and fathers is noticeably impaired. A considerable proportion of both mothers (30.3%) and fathers (31.1%) rated their own child’s overall mental health as impaired.

### Association of psychosocial variables between children with rare diseases, their mothers, and fathers

Bivariate correlation between outcome variables showed that mothers QoL was significantly associated with fathers QoL (*r* = 0.449, *p* < 0.001) and parent-reported HRQoL of their child (*r* = 0.232, *p* = 0.015). Fathers in contrast showed no significant association between their own QoL and parent-reported HRQoL of their child (*r* = 0.038, *p* = 0.705). In contrast, mothers perceived mental health was not significantly associated with fathers perceived mental health (*r* = 0.196, *p* = 0.055). However, parent-reported mental health of their child was significantly associated with their own perceived mental health in mothers (*r* = 0.312, *p* = 0.001) and fathers (*r* = 0.318, *p* = 0.001).

### Predictors for psychosocial outcomes in families of children with rare diseases

Table 3 shows multiple regression models with all predictors of psychosocial outcomes for mothers, fathers, and their children. The analyses revealed that younger age was associated with fewer parent-reported psychosocial problems on HRQoL of mothers and fathers and mental health of mothers. Being male was also associated with fewer impairment in parent-reported overall mental health in mothers. Lower education of mothers was significantly associated with lowered QoL in mothers. The presence of a level of care was significantly associated with more impairment in mothers’ mental health, parent-reported HRQoL of mothers and fathers, and parent-reported mental health of mothers. A shorter time interval to the last surgery also was found to be a significant predictor of parent-reported HRQoL of fathers.

**Table 3:** Prediction of psychosocial measures on children with rare congenital surgical diseases and their parents

	Constant		Age		Gender		Education		Level of care		Time since last operation		Adjusted R^2^
	*b*	95% CI	*b*	95% CI	*b*	95% CI	*b*	95% CI	*b*	95% CI	*b*	95% CI	
Mothers													
QoL	**2.34**	**[2.19, 2.53]**	0.01	[− 0.01, 0.03]	0.01	[− 0.12, 0.14]	− **0.16**	**[**− **0.29, **− **0.02]**	− 0.05	[− 0.17, 0.07]	0.01	[− 0.22, 0.04]	0.042
Mental health	**0.27**	**[0.01, 0.53]**	0.01	[− 0.03, 0.04]	0.09	[− 0.12, 0.29]	0.15	[− 0.06, − .36]	**0.20**	**[0.01, 0.39]**	− 0.02	[− 0.07, 0.03]	0.033
Children’s HRQoL	**90.63**	**[83.88, 97.39]**	− **1.03**	**[**− **1.93, **− **0.13]**	− 0.81	[− 6.02, 4.41]	− 1.51	[− 6.89, 3.87]	− **9.30**	**[**− **14.29, **− **4.31]**	0.68	[− 0.54, 1.89]	0.150
Children’s mental health	**9.85**	**[8.09, 11.61]**	**0.33**	**[0.09, 0.57]**	− **1.47**	**[**− **2.83, **− **0.11]**	0.39	[− 1.01, 1.80]	**1.39**	**[0.08, 2.69]**	− 0.21	[− 0.53, 0.11]	0.159
Fathers													
QoL	**2.36**	**[2.21, 2.51]**	0.01	[− 0.02, 0.03]	0.05	[− 0.08, 0.18]	− 0.11	[− 0.23, 0.02]	− 0.01	[− 0.14, 0.12]	0.01	[− 0.03, 0.04]	0.008
Mental health	**0.23**	**[0.09, 0.38]**	− 0.01	[− 0.03, 0.03]	0.01	[− 0.12, 0.12]	0.11	[− 0.01, − .23]	− 0.01	[− 0.12, 0.12]	− 0.01	[− 0.04, 0.02]	0.005
Children’s HRQoL	**90.01**	**[84.68, 95.34]**	− **1.58**	**[**− **2.59, **− **0.58]**	0.13	[− 4.39, 4.66]	− 0.13	[− 4.52, 4.26]	− **5.95**	**[**− **10.36, **− **1.53]**	**1.67**	**[0.42, 2.92]**	0.139
Children’s mental health	**11.24**	**[9.18, 13.30]**	0.26	[− 0.12, 0.65]	− 0.29	[− 2.04, 1.46]	0.19	[− 1.50, 1.89]	− 0.48	[− 2.18, 1.23]	− 0.44	[− 0.92, 0.04]	0.012

Bold values indicate statistical significance at the *p* < .05 level

Gender of child: female = 1, male = 0. Education: higher = 1, lower-middle = 0. Level of care: yes = 1, no = 0

*CI* confidence interval, *QoL* quality of life, *HRQoL* health-related quality of life

## Discussion

The COVID-19 pandemic has been particularly challenging for healthcare systems worldwide, while patients with rare diseases faced specific difficulties [5]. Even in stable times, patients with rare diseases and their families face care deficits and less than optimal medical support [46]. Although great strides have been made worldwide in addressing this unmet need, there was a considerable setback with the COVID-19 pandemic [6]. The study suggests that the COVID-19 pandemic and the lockdown measures may affect the psychosocial situation of families with rare congenital surgical diseases. The results concerning the negative impact of COVID-19 measures on HR-/QoL and mental health of parents [47] and their children [28, 47–55] are in line with recent studies from China, India, Bangladesh, Brazil, the US, Spain, Italy, and Germany [33, 47–54, 56].

Findings indicate that mothers and fathers of children with rare congenital surgical diseases had significantly lower overall QoL than parents in the control group. These findings may reflect the particular burden placed on the parents of children with rare congenital surgical diseases.

While many parents may face a burden by the daily care of their children and working from home and thus having less time to themselves due to the COVID-19 pandemic, parents of children with rare congenital diseases may experience a particular additional burden of having to care for their ill child and its consequences, such as being at high risk for infection and the particular need of care [49, 55]. Regarding parental mental health, mothers in the index group reported significantly higher overall mental impairment than mothers in the control group, which is in line with previous research on the mental health of parents with chronically ill children [57] and are consistent with the additional burden of the COVID-19 pandemic already described. Fathers in the index and control group showed no significant difference regarding mental health. In this regard, the results showed that mothers in the index group had significantly higher mental health impairment than their male counterparts. These results are supported by the findings that one-third of the mothers showed clinically significant levels of psychological impairment, whereas only about one-tenth of the fathers did. These gender differences in the index group were also found for QoL, with mothers having significantly lower QoL than fathers. These gender-specific differences in parental QoL and mental health are consistent with the current literature [23, 57, 58] and with the fact that mothers are more likely to be the primary caregiver and are thus more involved in the child’s care, which can be stressful and may lead to impairment in mental health and QoL [20, 59].

Regarding the children’s overall HRQoL, mothers and fathers in the index group reported significantly lower HRQoL of their children than the control group, which is in line with previous findings [24, 26]. A more nuanced view of the HRQoL subscales showed that more than a third of the mothers and fathers in the index group rated their children’s emotional functioning as strongly impaired.

Furthermore, both mothers and fathers in the index group reported significantly higher impairment regarding the children’s overall mental health than the control group. Although a previous study could show that children’s mental health appears to be considerably impaired during the COVID-19 pandemic [33], it seems that children with rare diseases experience additional impairment. This result may reflect that coping with the current situation and the impact of its restrictions by lockdown, social distancing, and homeschooling measures on their daily lives can be particularly difficult for all children, but especially for rare diseased children. This may be supported by the finding that about a third of mothers and fathers rated the overall mental health of their affected child as impaired. Thus, these results mostly confirm our first hypothesis on psychosocial outcomes of affected parents and their children within the framework of the diathesis-stress model [17].

Further, we compared our results to norm data collected before COVID-19, showing mothers and fathers in the index group also had significantly lower overall QoL than parents of children with chronic conditions such as diabetes or epilepsy. This reduced parental QoL is in line with a recent systematic review on the QoL of parents caring for children with rare diseases compared to parents of healthy children and norm values [20]. As with the control group referring to mental health, only mothers and not fathers were significantly impaired compared to norm data.

Concerning the children’s emotional and behavioral problems, the parent-reported overall HRQoL and mental health of their children was significantly reduced in the index group compared to the normative sample, which is consistent with previous research on HRQoL [13] and mental health in children with rare diseases. Studies that surveyed the rare congenital surgical diseases using the same instruments before the COVID-19 pandemic reported similar results on the children's HRQoL and mental health, thus approximately reflecting the “COVID-19 Yes–No” intervention variable [24–27]. Therefore, one possible explanation for the barely relevant difference to studies before the COVID-19 pandemic could be that children with rare congenital pediatric surgical diseases are already severely impaired, and the restrictions due to the pandemic may not have an additional impact on their psychosocial well-being. In contrast, conclusions about parents cannot be made due to the lack of comparable studies before the COVID-19 pandemic. Therefore, our second hypothesis is only partly confirmed.

Moreover, we looked for associations between the psychosocial outcomes of affected children, their mothers, and their fathers. Whereas maternal and paternal QoL were moderately associated, this was not the case for mental health. A possible explanation for the different results of the constructs could be that the parent’s QoL refers predominantly to the burden and impact of the child’s disease on daily life due to care responsibilities, whereas this applies less to the construct of mental health. In contrast, while mother’s and father’s mental health were moderately associated with the parent-reported mental health of their child, only very small to small associations could be found for the QoL of mothers and fathers and their parent-reports on HRQoL of their child. These findings may be explained by the fact that parental mental health more reflects the parent-reported mental health of their child, while this is less the case for parental QoL and parent-reported HRQoL. Overall, these findings partly confirm our third hypothesis.

Our final aim was to identify predictors of the respective psychosocial outcome variable of children with a rare congenital surgical disease and their parents. The younger age of the children was a significant predictor of parent-reported HRQoL, which is in contrast to the commonly favored presumption that most clinical sequelae resolve with age. However, previous studies have also reported better HRQoL in younger ages on rare congenital surgical diseases [60]. In addition, younger age and being male were significant predictors of higher parent-reported overall mental health in mothers. Moreover, less time since the last operation as well as receiving a level of care were identified as risks for higher impairment in the children’s HRQoL reported by fathers, whereas in mothers, only receiving a level of care was found to be a predictor for the children’s HRQoL, while also being a significant risk factor for their own and mother-reported mental health. These findings correspond to the fact that the presence of a level of care is associated with higher disease severity, which may influence the child’s and their parent’s well-being and has already been shown in previous research [24, 60, 61].

Further, parental education was identified as a significant predictor of QoL in mothers, with lower QoL associated with lower education, which is in line with a previous study on a pediatric population [61]. Although many families were facing financial hardship due to the pandemic, this may be especially the case for families with lowered socioeconomic status, which may be reflected by the lowered parental QoL. Thus, our fourth hypothesis is partly confirmed by our findings.

### Study limitations

The present study was subject to several limitations: (1) our study was conducted over 12 months due to the inevitable delay of ethical approval and difficulties in recruiting. Therefore, our results may be confined to implications of whether there was a partial lockdown taking place in Germany. Post-hoc comparison of all psychosocial variables of whether a partial lockdown took place revealed no significant differences. (2) We cannot rule out the possibility of a non-response bias. The affected children of participating families were significantly younger compared to affected children of non-participating families. One might argue that older children are less affected by their disease, and parents may not have less relevance in participating. Therefore, the psychosocial impairment of affected families might be overestimated. Nevertheless, the participation rate was similar to previous studies examining child health in Europe and the US [33, 62]. (3) Even though the heterogeneity of rare diseases in this sample may be limiting, with the only consistent characteristic being the pediatric conditions requiring surgical treatments, no significant differences between disease groups were found. Thus, the findings might represent comparable disease groups, all of which are associated with a particularly high need for care and a high level of disease management. (4) Since only normative values and a narrative comparison were available for the “COVID-19 Yes–No” variable, it impossible to make reliable conclusions about main and interaction effects on the respective outcomes due to the COVID-19 pandemic. (5) Due to the pandemic numerous hospitals limited their acceptance to only emergent cases, which may have resulted in the cancellation or postponement of scheduled procedures for children with rare congenital surgical diseases. Although this was not the case for this study, this may limit the generalizability of the current results. (6) All families were recruited in northern Germany. Thus, a transfer of results to countries with different health care systems and COVID-19 prevention measures should be done with caution.

## Conclusion

With a large sample of families of children with rare congenital surgical diseases, our study highlights the considerable psychosocial impairment perceived by parents, especially among mothers, during the COVID-19 pandemic in Germany. Besides the fact that the COVID-19 pandemic is stressful for parents themselves, our results show that it also negatively affects the affected children. Health care professionals should be aware of the impact of caring for a rare diseased child on parental QoL and mental health. Family-oriented programs, especially in the pandemic, should be available for families with rare diseased children and other pediatric disease populations [63].

## Supplementary Information

**Additional file 1**. Distribution of familial psychosocial variables for the index group, the control group, and norm data.

## Data Availability

The datasets generated during the current study are available from the corresponding author on reasonable request.

## Abbreviations

HRQoL: Health related quality of life
QoL: Quality of life
COVID-19: Coronavirus disease 2019
ULQIE: Ulm Quality of Life Inventory for Parents
BSI: The Brief Symptom Inventory
PedsQL™ 4.0 SF15: The Pediatric Quality of Life Inventory™ Short Form 15
SDQ: The Strengths and Difficulties Questionnaire
