# Supplementary material for: Quality of life and mental health of children with rare congenital surgical diseases and their parents during the COVID-19 pandemic
Source: Orphanet J Rare Dis. 2021 Nov 27;16:498. doi: 10.1186/s13023-021-02129-0 (PMC8626760; doi:10.1186/s13023-021-02129-0)
Supplement: Supplementary file 1 — Additional file 1. Distribution of familial psychosocial variables for the index group, the control group, and norm data. [file 13023_2021_2129_MOESM1_ESM.docx]

**Table S1.** Distribution of parental QoL for the index group, the control group, and norm data of the ULQIE

|  |  | **Index group (a)** | |  | **Control group (b)** | |  | **Norm data (c)** | | **Differences** | **Effect size**  **a vs. b** | **Effect size**  **a vs. c** |
| --- | --- | --- | --- | --- | --- | --- | --- | --- | --- | --- | --- | --- |
|  |  | ***M*** | ***SD*** |  | ***M*** | ***SD*** |  | ***M*** | ***SD*** |  |  |  |
| Mothers |  |  |  |  |  |  |  |  |  |  |  |  |
| Physical & daily functioning |  | 2.5 | 0.41 |  | 2.7 | 0.54 |  | 2.6 | 0.70 | a < b*; a < c | -0.44 | -0.17 |
| Satisfaction with family |  | 3.2 | 0.66 |  | 3.4 | 0.57 |  | 3.2 | 0.67 | a < b*; a < c | -0.38 | -0.10 |
| Emotional stability |  | 1.9 | 0.68 |  | 2.4 | 0.98 |  | 2.5 | 0.75 | a < b**; a < c*** | -0.56 | -0.81 |
| Self-development |  | 1.5 | 0.71 |  | 1.6 | 0.89 |  | 2.1 | 0.73 | a < b; a < c*** | -0.04 | -0.80 |
| Well-being |  | 2.4 | 0.50 |  | 2.7 | 0.70 |  | 2.7 | 0.75 | a < b**; a < c*** | -0.58 | -0.70 |
| Total score |  | 2.3 | 0.31 |  | 2.6 | 0.52 |  | 2.6 | 0.53 | a < b***; a < c*** | -0.82 | -1.17 |
| Fathers |  |  |  |  |  |  |  |  |  |  |  |  |
| Physical & daily functioning |  | 2.6 | 0.43 |  | 2.9 | 0.63 |  | 2.6 | 0.70 | a < b**; a > c | -0.66 | 0.05 |
| Satisfaction with family |  | 3.4 | 0.56 |  | 3.5 | 0.57 |  | 3.2 | 0.67 | a < b; a > c | -0.30 | 0.20 |
| Emotional stability |  | 1.7 | 0.65 |  | 2.6 | 1.15 |  | 2.5 | 0.75 | a < b***; a < c*** | -1.11 | -1.20 |
| Self-development |  | 1.9 | 0.84 |  | 1.8 | 0.76 |  | 2.1 | 0.73 | a > b; a < c* | 0.12 | -0.23 |
| Well-being |  | 2.5 | 0.47 |  | 2.9 | 0.63 |  | 2.7 | 0.73 | a < b***; a < c*** | -0.79 | -0.40 |
| Total score |  | 2.4 | 0.30 |  | 2.8 | 0.60 |  | 2.6 | 0.53 | a < b***; a < c*** | -1.02 | -0.96 |

Note. Raw scores of the ULQIE, with higher values corresponding to higher parental QoL. Norm data of parents of children with chronic conditions such as diabetes and epilepsy, self-report (Goldbeck & Storck, 2002). Comparison between groups is assessed with Welch *t*-test and one-sample *t*-test. * *p* ≤ 0.05, ** *p* ≤ 0.01, *** *p* ≤ 0.001, *d* = Cohen’s *d.*

**Table S2.** Distribution of parental mental healthfor the index group, the control group, and norm data of the BSI

|  |  | **Index group (a)** | | **Control group (b)** | |  | **Norm data (c)** | | **Differences** | **Effect size**  **a vs. b** | **Effect size**  **a vs. c** |
| --- | --- | --- | --- | --- | --- | --- | --- | --- | --- | --- | --- |
|  |  | ***M*** | ***SD*** | ***M*** | ***SD*** |  | ***M*** | ***SD*** |  |  |  |
| Mothers |  |  |  |  |  |  |  |  |  |  |  |
| Somatization |  | 0.4 | 0.51 | 0.2 | 0.23 |  | 0.3 | 0.33 | a > b**; a > c | 0.43 | 0.12 |
| Obsessive-compulsive |  | 0.8 | 0.75 | 0.5 | 0.50 |  | 0.5 | 0.43 | a > b**; a > c** | 0.41 | 0.29 |
| Interpersonal sensitivity |  | 0.6 | 0.75 | 0.3 | 0.45 |  | 0.5 | 0.45 | a > b**; a > c | 0.39 | 0.15 |
| Depression |  | 0.5 | 0.57 | 0.3 | 0.33 |  | 0.3 | 0.40 | a > b**; a > c* | 0.40 | 0.20 |
| Anxiety |  | 0.4 | 0.53 | 0.3 | 0.33 |  | 0.4 | 0.36 | a > b*; a > c | 0.32 | 0.05 |
| Hostility |  | 0.6 | 0.60 | 0.4 | 0.36 |  | 0.3 | 0.33 | a > b**; a > c*** | 0.39 | 0.34 |
| Phobic anxiety |  | 0.3 | 0.50 | 0.2 | 0.36 |  | 0.2 | 0.25 | a > b; a > c* | 0.13 | 0.23 |
| Paranoid ideation |  | 0.5 | 0.63 | 0.3 | 0.40 |  | 0.3 | 0.38 | a > b*; a > c** | 0.37 | 0.25 |
| Psychoticism |  | 0.3 | 0.55 | 0.2 | 0.34 |  | 0.2 | 0.27 | a > b*; a > c** | 0.33 | 0.25 |
| Global Severity Index |  | 0.5 | 0.48 | 0.3 | 0.24 |  | 0.4 | 0.23 | a > b***; a > c** | 0.52 | 0.27 |
| Fathers |  |  |  |  |  |  |  |  |  |  |  |
| Somatization |  | 0.2 | 0.32 | 0.2 | 0.24 |  | 0.2 | 0.31 | a > b; a < c | 0.10 | -0.13 |
| Obsessive-compulsive |  | 0.5 | 0.51 | 0.4 | 0.58 |  | 0.5 | 0.56 | a > b; a < c | 0.15 | -0.07 |
| Interpersonal sensitivity |  | 0.3 | 0.45 | 0.3 | 0.48 |  | 0.3 | 0.40 | a > b; a < c | 0.15 | -0.05 |
| Depression |  | 0.2 | 0.47 | 0.3 | 0.61 |  | 0.2 | 0.32 | a < b; a < c | -0.02 | -0.03 |
| Anxiety |  | 0.2 | 0.32 | 0.2 | 0.35 |  | 0.3 | 0.31 | a > b; a < c | 0.06 | -0.18 |
| Hostility |  | 0.3 | 0.37 | 0.3 | 0.43 |  | 0.3 | 0.35 | a > b; a > c | 0.13 | 0.14 |
| Phobic anxiety |  | 0.2 | 0.28 | 0.2 | 0.30 |  | 0.1 | 0.23 | a < b; a > c | -0.07 | 0.05 |
| Paranoid ideation |  | 0.3 | 0.41 | 0.3 | 0.51 |  | 0.3 | 0.40 | a > b; a < c | 0.02 | -0.05 |
| Psychoticism |  | 0.1 | 0.26 | 0.2 | 0.47 |  | 0.2 | 0.28 | a < b; a < c* | -0.12 | -0.22 |
| Global Severity Index |  | 0.3 | 0.28 | 0.3 | 0.37 |  | 0.3 | 0.23 | a > b; a < c | 0.07 | -0.05 |

Note. *S*um scores are given, with higher scores indicating higher psychological distress. Global Severity Index = sum of all items of the Brief Symptom Inventory. Norm data of healthy adults separated by gender, self-report (Franke, 2000). Comparison between groups is assessed with Welch *t*-test and one-sample *t*-test. * *p* < 0.05, ** *p* < 0.01, *** *p* < 0.001. *d =* Cohen’s *d.*

**Table S3.** Distribution of parent-reported HRQoL of children and adolescents the index group, the control group, and norm data of the PedsQL-SF 15

|  |  | **Index group (a)** | |  | **Control group (b)** | | |  | **Norm data (c)** | | | **Differences** | | **Effect size a vs. b** | **Effect size a vs. c** |
| --- | --- | --- | --- | --- | --- | --- | --- | --- | --- | --- | --- | --- | --- | --- | --- |
|  |  | ***M*** | ***SD*** |  | | ***M*** | ***SD*** |  | | ***M*** | ***SD*** | |  |  |  |
| Mothers |  |  |  |  | |  |  |  | |  |  | |  |  |  |
| Physical Health |  | 87.0 | 19.62 |  | | 94.6 | 11.13 |  | | 91.7 | 10.7 | | a < b**; a < c | -0.44 | -0.24 |
| Psychosocial Health |  | 78.8 | 13.96 |  | | 84.0 | 12.30 |  | | 83.3 | 13.7 | | a < b*; a < c* | -0.39 | -0.32 |
| Emotional functioning |  | 72.3 | 18.94 |  | | 74.9 | 17.99 |  | | 81.2 | 17.0 | | a < b; a < c*** | -0.14 | -0.47 |
| Social functioning |  | 88.0 | 14.55 |  | | 90.4 | 11.29 |  | | 86.9 | 16.8 | | a < b; a > c | -0.17 | 0.07 |
| School functioning |  | 78.2 | 21.48 |  | | 89.3 | 17.73 |  | | 82.1 | 19.6 | | a < b***; a < c | -0.54 | -0.18 |
| Total Score |  | 81.5 | 13.79 |  | | 87.7 | 9.80 |  | | 86.1 | 11.2 | | a < b***; a < c** | -0.49 | -0.33 |
| Fathers |  |  |  |  | |  |  |  | |  |  | |  |  |  |
| Physical Health |  | 87.6 | 14.27 |  | | 97.2 | 5.16 |  | | 91.7 | 10.7 | | a < b**; a > c | -0.42 | 0.04 |
| Psychosocial Health |  | 80.0 | 12.99 |  | | 87.9 | 11.21 |  | | 83.3 | 13.7 | | a < b**; a < c | -0.56 | -0.18 |
| Emotional functioning |  | 72.3 | 17.67 |  | | 81.1 | 14.75 |  | | 81.2 | 17.0 | | a < b**; a < c*** | -0.52 | -0.50 |
| Social functioning |  | 90.2 | 12.38 |  | | 92.4 | 10.08 |  | | 86.9 | 16.8 | | a < b; a > c** | -0.18 | 0.27 |
| School functioning |  | 82.4 | 19.54 |  | | 90.9 | 16.15 |  | | 82.1 | 19.6 | | a < b*; a > c | -0.46 | 0.01 |
| Total Score |  | 85.0 | 11.91 |  | | 91.2 | 8.37 |  | | 86.1 | 11.2 | | a < b***; a < c | -0.56 | -0.09 |

Note. The possible range for subscales 0-100. Higher scores indicate better HRQoL. Norm data of healthy children and adolescents, parent-report (Chan et al., 2005). Comparison between groups is assessed with Welch *t*-test and one-sample *t*-test. * *p* < 0.05, ** *p* < 0.01, *** *p* < 0.001. *d =* Cohen’s *d*.

**Table S4.** Distribution of parent-reported mental health of children and adolescents the index group, the control group, and norm data of the SDQ

|  |  | **Index group (a)** | |  | **Control group (b)** | |  | **Norm data (c)** | | **Differences** | **Effect size a vs. b** | | **Effect size a vs. c** | |
| --- | --- | --- | --- | --- | --- | --- | --- | --- | --- | --- | --- | --- | --- | --- |
|  |  | ***M*** | ***SD*** |  | ***M*** | ***SD*** |  | ***M*** | ***SD*** |  | |  | |  |
| Mothers |  |  |  |  |  |  |  |  |  |  | |  | |  |
| Emotional symptoms |  | 2.0 | 1.98 |  | 1.5 | 1.61 |  | 2.0 | 2.41 | a > b; a < c | | 0.24 | | -0.01 |
| Conduct problems |  | 2.5 | 1.45 |  | 2.3 | 1.13 |  | 2.2 | 2.41 | a > b; a > c* | | 0.21 | | 0.22 |
| Hyperactivity |  | 4.6 | 1.68 |  | 5.3 | 1.22 |  | 3.2 | 3.61 | a < b**; a > c*** | | -0.45 | | 0.83 |
| Peer problems |  | 2.2 | 1.81 |  | 1.1 | 1.39 |  | 1.4 | 2.41 | a > b***; a > c*** | | 0.66 | | 0.43 |
| Prosocial behavior |  | 6.5 | 2.95 |  | 7.6 | 2.17 |  | 8.3 | 2.41 | a < b*; a < c*** | | -0.39 | | -0.60 |
| Total |  | 11.3 | 3.65 |  | 10.1 | 2.96 |  | 8.5 | 7.22 | a > b*; a > c*** | | 0.33 | | 0.75 |
| Fathers |  |  |  |  |  |  |  |  |  |  | |  | |  |
| Emotional symptoms |  | 1.5 | 1.83 |  | 1.2 | 1.82 |  | 2.0 | 2.41 | a > b; a < c** | | 0.20 | | -0.23 |
| Conduct problems |  | 2.4 | 1.32 |  | 2.2 | 1.17 |  | 2.2 | 2.41 | a > b; a > c | | 0.16 | | 0.09 |
| Hyperactivity |  | 5.0 | 1.86 |  | 5.5 | 1.40 |  | 3.2 | 3.61 | a < b; a > c*** | | -0.27 | | 0.98 |
| Peer problems |  | 2.3 | 2.20 |  | 1.5 | 1.29 |  | 1.4 | 2.41 | a > b*; a > c** | | 0.38 | | 0.39 |
| Prosocial behavior |  | 6.6 | 2.93 |  | 7.4 | 2.72 |  | 8.3 | 2.41 | a < b; a < c*** | | -0.29 | | -0.58 |
| Total |  | 11.1 | 4.01 |  | 10.2 | 3.53 |  | 8.5 | 7.22 | a > b; a > c*** | | 0.24 | | 0.65 |

Note. The possible range for subscales 0-10 and total score 0-40. Higher scores represent lower mental health for all subscales, except prosocial behavior, where lower scores correspond to more difficulties in prosocial behavior. Norm data of children and adolescents, parent-report (Hölling et al., 2014). *d =* Cohen’s *d.* * *p* < 0.05, ** *p* < 0.01, *** *p* < 0.001.
